# ﻿Two new species of larval Erythraeidae (Parasitengona) ectoparasites of leafhoppers from Southwestern China

**DOI:** 10.3897/zookeys.1236.139274

**Published:** 2025-04-25

**Authors:** Yan Jiang, Tian-Ci Yi, Si-Yuan Xu, Dao-Chao Jin

**Affiliations:** 1 Institute of Entomology, Guizhou University, Guizhou Provincial Key Laboratory for Plant Pest Management of the Mountainous Region, the Scientific Observing and Experimental Station of Crop Pest in Guiyang, Ministry of Agriculture and Rural Affairs of the P. R. China, Guiyang 550025, China Guizhou University Guiyang China; 2 Department of Pharmacy, Guizhou Provincial Engineering Research Center of Medical Resourceful Healthcare Products, Guiyang Healthcare Vocational University, Guiyang 550000, China Guiyang Healthcare Vocational University Guiyang China

**Keywords:** Abrolophinae, Callidosomatinae, Chongqing, insect, new host, range extension, taxonomy, Yunnan

## Abstract

In this study, three species were examined. Among them, two new species, *Caeculisomataianensis***sp. nov.** and *Iguatoniabarboproxima***sp. nov.** from Southwestern China, were described and illustrated based on larvae. The two new species can be distinguished from the known species by the following characteristics: the anterior sensilla are nude, and the gnathosoma has two pairs of nude hypostomalae in *C.taianensis***sp. nov.**; the anterior sensilla and posterolateral scutalae are located in the posterior half of the scutum, and the posterior hypostomalae with barbs on the proximal half in *I.barboproxima***sp. nov.** In addition, a new host and distribution range of *Abrolophusquadrapexicis* Xu & Jin, 2022 is reported.

## ﻿Introduction

Southwestern China comprises three provinces (Sichuan, Guizhou, and Yunnan), one municipality (Chongqing), and one autonomous region (Xizang (Tibet)). This region is characterized by diverse topography with significant variations in elevation and numerous separated basins (Shi et al. 2018), including the Yunnan-Guizhou Plateau, the Qinghai-Tibetan Plateau, the Hengduan Mountains, and the Sichuan Basin. Simultaneously, the region exhibits diverse climatic conditions, including a subtropical monsoon climate, plateau mountain climate, mountain climate, tropical monsoon climate, dry-hot valley climate and a temperate monsoon climate. The variety of climates provides a wealth of environmental conditions, which play a pivotal role in fostering species diversity in the ecosystem. Therefore, Southwestern China is a key area for fauna-flora biodiversity research in China (Han et al. 2008; Shi et al. 2018).

To date, 13 species in eight genera (*Balaustium* von Heyden, 1826, *Caeculisoma* Berlese, 1888, *Charletonia* Oudemans, 1910, *Erythraeus* Latreille, 1806, *Grandjeanella* Southcott, 1961, *Leptus* Latreille, 1796, *Marantelophus* Haitlinger, 2011, and *Neoabrolophus* Khot, 1965) of five subfamilies (Abrolophinae Witte, 1995, Balaustiinae Grandjean, 1947, Callidosomatinae Southcott, 1957, Erythraeinae Robineau-Desovidy, 1828, and Leptinae Billberg, 1820) of Erythraeidae have been documented in Southwestern China (Xu et al. 2017, 2019a, b, 2020, 2022a, 2022b, 2023, 2025). Of them, three species were described based on post-larval forms, one species was recorded based on both larval and post-larval stages, and the remaining species were known only by larvae (Table 1).

**Table 1.** Hosts and distribution of the known species of Erythraeidae from Southwestern China.

Species	Host	Distribution
*Balaustiumallomedicagoense* Xu & Jin, 2025 [P]	Unknown	Yunnan
*Balaustiumneomedicagoense* Xu & Jin, 2025 [P]	Unknown	Xizang
*Caeculisomapenlineatus* Xu & Jin, 2019 [L]	*Mileewamargheritae* (female) (Hemiptera, Cicadellidae, Mileewinae), *Neuterthronhamuliferum* (male) (Hemiptera, Delphacidae), unidentified Alebrini (Hemiptera, Cicadellidae, Typhlocybinae), unidentified Delphacinae (Hemiptera, Delphacidae), unidentified Issidae (Hemiptera, Auchenorrhyncha)	Chongqing, Guizhou
*Caeculisomasemispinus* Xu & Jin, 2019 [L]	*Shaddai* sp. (male) (Hemiptera, Cicadellidae), unidentified Zyginellini (female) (Cicadellidae)	Chongqing
*Charletoniarectangia* Xu & Jin, 2022 [L]	Unidentified Acrididae (Orthoptera), unidentified Tettigoniidae (Orthoptera), unknown Chrysomelidae (Coleoptera), unidentified mantis (Mantodea), unidentified moth (Lepidoptera), unidentified stick insect (Phasmatodea)	Yunnan
Erythraeus (Zaracarus) mossesus Xu & Jin, 2023 [P, L]	Unknown	Guizhou
*Grandjeanelladianensis* Xu & Jin, 2022 [L]	Unknown.	Yunnan
Leptus (Leptus) bomiensis Xu & Jin, 2022 [L]	Unidentified moth (Lepidoptera), unidentified Elateridae (Coleoptera), unidentified Pentatomidae (Hemiptera)	Xizang
Leptus (Leptus) striatus Xu & Jin, 2022 [L]	Unidentified Opiliones	Yunnan
Leptus (Leptus) trisolenidionus Xu & Jin, 2022 [L]	Unidentified Cicadellinae (Hemiptera, Cicadellidae)	Guizhou
*Marantelophusdubifurcatus* Xu, Yi & Jin, 2017 [L]	*Cacopsylla* sp. (Hemiptera, Psyllidae), unidentified Psocoptera.	Guizhou
*Marantelophusneodubifurcatus* Xu & Jin, 2023 [L]	Unknown	Guizhou
*Neoabrolophusguizhouensis* Xu & Jin, 2025 [P]	Unknown	Guizhou

In this checklist, host and distribution data were obtained from Xu et al. 2017, 2019a, b, 2020, 2022a, b, 2023, 2025. [P]: Post-larval form. [L]: Larva.

Species of the genus *Abrolophus* Berlese, 1891 from China are distributed in the Macao Special Administrative Region, Hainan Province, Zhejiang Province, Shandong Province, Guangxi Zhuang Autonomous Region, Guangdong Province, and Hunan Province (Zheng 2002a; Haitlinger 2006; Xu et al. 2021, 2022a). However, there has been no report of *Abrolophus* in Southwestern China.

A total of 27 species of *Caeculisoma* have been reported worldwide, of which 14 were based on the post-larval stage, 12 were based on the larval stage, and only one species was based on both larval and post-larval instars (Mąkol and Wohltmann 2012; Xu et al. 2019a, 2019b, 2020; Saboori et al. 2023; Kohansal et al. 2024; Noei et al. 2024). Among the 27 known *Caeculisoma* species, four were recorded in China based on the larval stage, with two (*C.penlineatus* Xu & Jin, 2019, *C.semispinus* Xu & Jin, 2019) collected from Southwestern China (Zheng 2002b; Xu et al. 2019a, 2019b, 2020).

Hitherto, only two species of the genus *Iguatonia* have been reported based on their larval stage worldwide (Haitlinger 2004; Xu et al. 2020; Noei et al. 2024): *I.barbillae* Haitlinger, 2004 from Brazil and *I.xinfengi* Xu & Jin, 2020 from Hainan Province (Island), China.

In this study, two new species, *C.taianensis* sp. nov. and *I.barboproxima* sp. nov., collected from Yunnan Province and Chongqing Municipality, respectively, are described and illustrated based on larvae. Additionally, new data for *A.quadrapexicis* Xu & Jin, 2022 is provided.

## ﻿Material and methods

Erythraeid larvae were collected along with their insect hosts using 200 mesh insect nets, and subsequently preserved in small vials containing absolute ethanol. The larvae on the hosts were detached using a fine brush under a stereomicroscope (Nikon SMZ745) in the lab. Then, all larval specimens were cleared in Oudemans’ fluid for about 12 h at 25 °C and slide-mounted in Hoyer’s medium (Walter and Krantz 2009). Figures were drawn with the aid of a drawing tube attached to a Nikon Eclipse Ni-E compound microscope. Genus identification is based on the key to world genera of larval Callidosomatinae of Noei et al. (2024). Terminology and abbreviations are adapted from Noei et al. (2024), Wohltmann et al. (2007) and Xu et al. (2020). Measurements are expressed in micrometers (μm). The standard deviations (SD) are provided with two decimal places. All specimens were deposited at the Institute of Entomology, Guizhou University, Guiyang, China (GUGC).

## ﻿Results

### ﻿Abrolophinae Witte, 1995

***Abrolophus* Berlese, 1891**

#### ﻿ Abrolophus quadrapexicis

Taxon classificationAnimaliaTrombidiformesErythraeidae

Xu & Jin, 2022

A26E19A0-AF53-5C5F-961E-59122D327E44

##### Material examined.

China • one larva (2000–1600-GZ-yj); Guizhou Province, Fanjingshan National Nature Reserve; 27°59'27″N, 108°33'30″E; 673 m; 30 Jun. 2023; Si-Yuan Xu leg.; on an unidentified Psyllidae (Hemiptera).

##### Distribution.

Guizhou Province (new distribution), Shandong Province, Zhejiang Province.

##### Note.

This species was collected from plants without a host record in Zhejiang and Shandong (Xu et al. 2022b).

### ﻿Callidosomatinae Southcott, 1957

***Caeculisoma* Berlese, 1888**

#### ﻿ Caeculisoma taianensis sp. nov.

Taxon classificationAnimaliaTrombidiformesErythraeidae

F0A460C9-EA62-55AC-BE81-E3CE2C1A1F06

https://zoobank.org/F5C1A34A-2366-423A-B816-969D2202B854

Figs 1 , 2 , 3 , 4

##### Diagnosis (larva).

ASE nude and posterior to the level of ML, closer to ML than PL; PSE with barbs on the distal one-third; gnathosoma with two pairs of nude hypostomalae; ISD 56–63; Ti I 188–207; Ti III 264–287.

##### Description.

**Figure 1.**
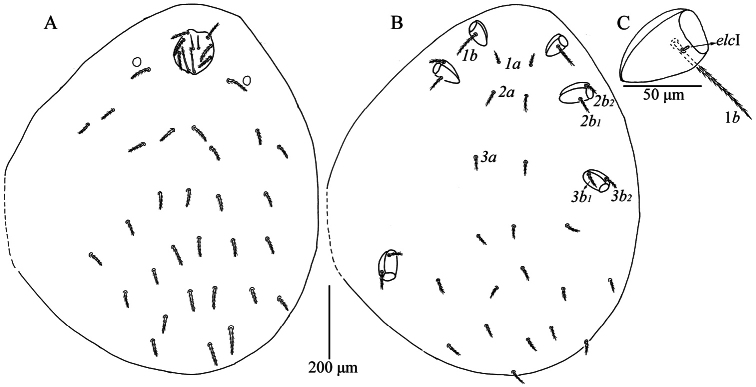
*Caeculisomataianensis* sp. nov., larva **A** dorsal view of idiosoma **B** ventral view of idiosoma **C** dorsal view of coxa I, showing supracoxal seta.

**Figure 2.**
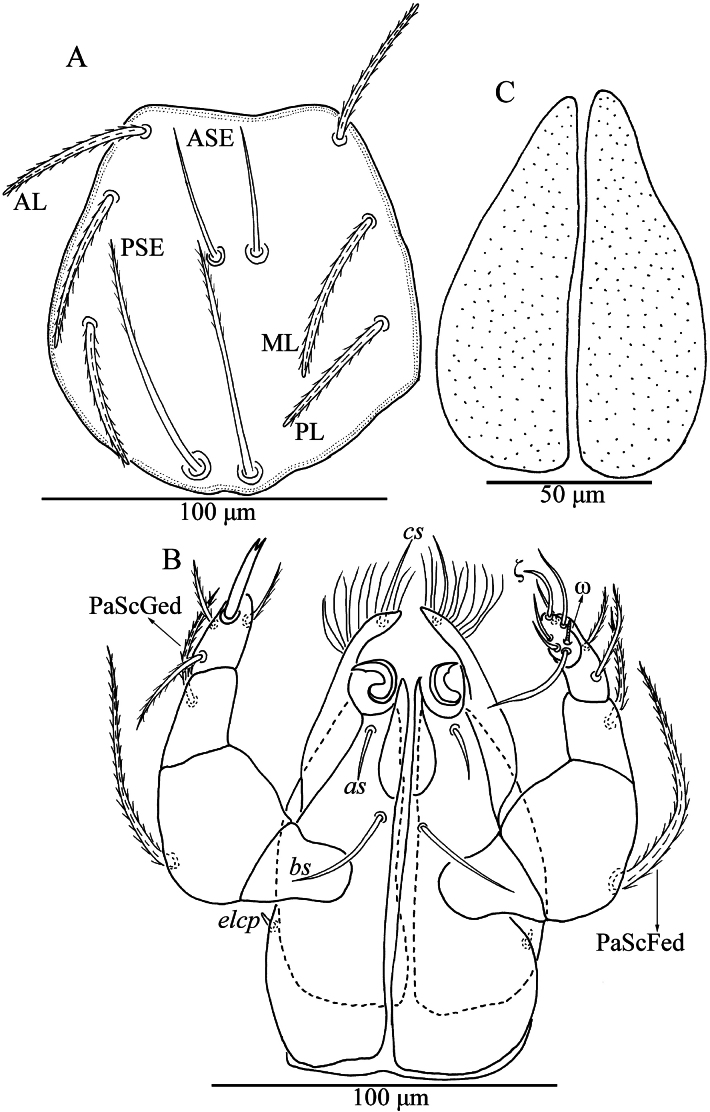
*Caeculisomataianensis* sp. nov., larva **A** scutum **B** ventral view of gnathosoma **C** dorsal view of the cheliceral bases.

**Figure 3.**
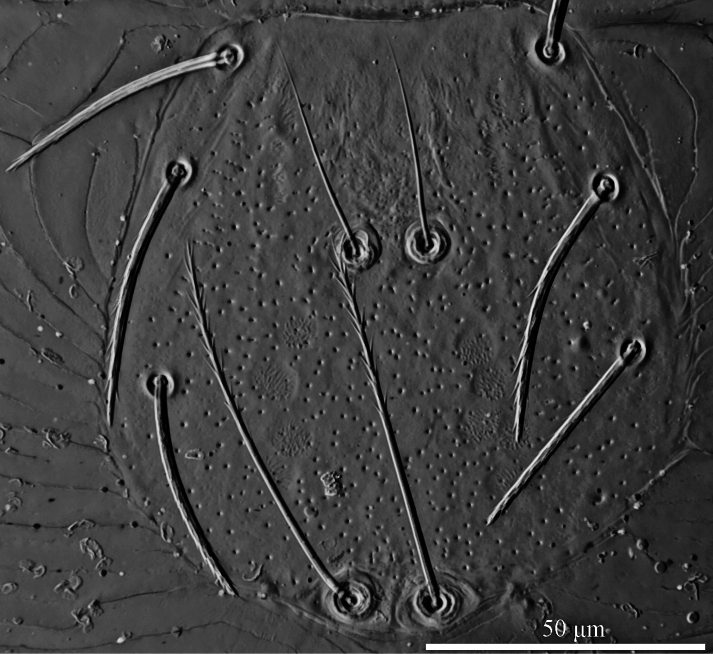
*Caeculisomataianensis* sp. nov., larva photograph. Scutum, showing the shape of scutum, ASE and PSE.

Dorsum. Idiosoma lateral cuticle of holotype used for drawing broken slide preparation, almost oval, with 30 (fD = 28–30 in paratypes) barbed setae, a pair of setae located between scutum and eyes (Fig. 1A). Scutum outline pentagonal with rounded angles, length somewhat longer than width, anterior margin slightly concave, anterolateral and posterolateral margins slightly sinuous, posterior margin with small concavity between bases of PSE (Figs 1A, 2A, 3). Three pairs of normal setae (AL, ML and PL), and two pairs of sensilla (ASE and PSE) placed on scutum. AL, ML and PL completely barbed, ASE nude, PSE with fine barbs in distal about one-third. ASE placed between ML and PL, and closer to ML than PL, PSE near posterior margin of scutum. PSE much longer than ASE, ML slightly longer than AL and PL, AL slightly longer than PL, one paratype (c) AL equal to PL (Table 2).

**Table 2.** Metric and some meristic data of *Caeculisomataianensis* sp. nov. (larvae, a–f = paratypes; a: 1088–0275-YN-yl, b: 1082–0271-YN-yl, c: 1083–0271-YN-yl, d: 1084–0271-YN-yl, e: 1085–0272-YN-yl, f: 1086–0273-YN-yl).

Character	Holotype	a	b	c	d	e	f	SD	range
fD	30	28	30	30	28	30	28	0.99	28–30
fV	14	14	12	14	12	14	14	0.90	12–14
NDV	44	42	42	44	40	44	42	1.40	40–44
IL	999	689	1121	901	1069	1061	1543	240.10	689–1543
IW	843	415	840	608	720	678	1028	181.47	415–1028
DS	30–66	33–57	33–59	32–62	36–64	33–60	33–55	1.64–3.58	30–66
DS min.	30	33	33	32	36	33	33	1.64	30–36
DS max.	66	57	59	62	64	60	55	3.58	55–66
PDS	34–66	36–57	37–59	34–62	37–64	36–60	33–55	1.48–3.58	33–66
PDS min.	34	36	37	34	37	36	33	1.48	33–37
PDS max.	66	57	59	62	64	60	55	3.58	55–66
Oc	21	22	22	23	21	20	19	1.25	19–23
*1a*	35	30	32	27	21	25	24	4.53	21–35
*2a*	44	47	43	38	39	44	40	3.00	38–47
*3a*	38	36	38	32	34	35	33	2.17	32–38
*1b*	74	67	74	66	72	64	71	3.73	64–74
*2b_1_*	47	49	43	44	47	48	40	2.97	40–49
*2b_2_*	36	34	29	32	33	34	31	2.12	29–36
*3b_1_*	48	44	46	44	46	46	40	2.36	40–48
*3b_2_*	35	37	36	36	34	34	32	1.55	32–37
L	114	109	109	110	111	114	103	3.46	103–114
W	108	103	103	100	103	102	97	3.10	97–108
AW	57	60	57	53	55	61	56	2.56	53–61
MW	76	71	71	71	74	72	69	2.14	69–76
PW	85	78	79	77	79	80	76	2.70	76–85
MA	41	38	40	43	40	43	38	1.92	38–43
AA	13	12	12	13	12	13	11	0.70	11–13
SB	15	14	15	14	15	16	14	0.70	14–16
ISD	63	60	60	57	59	58	56	2.14	56–63
AP	56	52	54	55	54	53	54	1.20	52–56
AL	46	45	37	44	44	45	40	3.02	37–46
ML	50	50	49	48	46	51	47	1.67	46–51
PL	41	41	37	37	37	36	35	2.19	35–41
ASE	39	34	33	39	31	36	33	2.88	31–39
PSE	71	66	60	70	60	67	57	5.04	57–71
*as*	15	14	16	15	17	18	16	1.25	14–18
*bs*	34	27	30	36	35	33	34	2.91	27–36
*cs*	26	23	20	28	25	22	25	2.47	20–28
PaScFed	74	78	76	81	83	85	73	4.24	73–85
PaScGed	36	33	31	34	37	36	34	1.92	31–37
GL	136	141	132	135	136	140	138	2.85	132–141
Ta I (H)	22	19	22	20	18	18	15	2.29	15–22
Ta I (L)	152	153	160	159	152	160	157	3.44	152–160
Ti I	199	191	200	192	201	207	188	6.22	188–207
Ge I	162	155	166	151	151	162	152	5.76	151–166
TFe I	93	88	93	88	92	96	89	2.81	88–96
BFe I	105	96	100	94	97	101	95	3.61	94–105
Tr I	54	47	48	48	47	44	52	3.11	44–54
Cx I	56	51	57	58	63	64	62	4.27	51–64
Ta II (H)	18	20	19	21	22	17	17	1.81	17–22
Ta II (L)	157	153	158	154	157	159	161	2.56	153–161
Ti II	215	198	214	204	211	216	194	8.17	194–216
Ge II	161	150	161	154	157	163	148	5.39	148–163
TFe II	95	86	85	85	91	90	84	3.78	84–95
BFe II	95	93	96	96	96	102	91	3.16	91–102
Tr II	58	54	50	53	48	51	54	3.02	48–58
Cx II	61	66	69	67	68	74	75	4.44	61–75
Ta III (H)	15	19	14	13	14	18	14	2.12	13–19
Ta III (L)	170	167	170	165	171	169	176	3.19	165–176
Ti III	287	266	277	264	271	277	273	7.17	264–287
Ge III	161	153	164	153	156	166	156	4.87	153–166
TFe III	136	124	132	133	128	131	126	3.89	124–136
BFe III	124	119	121	120	119	128	118	3.28	118–128
Tr III	66	57	57	53	53	54	51	4.61	51–66
Cx III	66	66	67	71	72	78	66	4.20	66–78
Leg I	821	781	824	790	803	834	795	18.28	781–834
Leg II	842	800	833	813	828	855	807	18.34	800–855
Leg III	1010	952	988	959	970	1003	966	20.68	952–1010
IP	2673	2533	2645	2562	2601	2692	2568	56.05	2533–2692

Venter. All ventral setae, including coxalae, barbed and with pointed ends (Fig. 1B). Three pairs of intercoxal setae (*1a*, *2a* and *3a*), *2a* longer than *1a* and *3a*, *3a* slightly longer than *1a* (Table 2), 14 setae behind coxae III (fV = 12–14 in paratypes). Five pairs of coxalae (*1b*, *2b_1_*, *2b_2_*, *3b_1_* and *3b_2_*), *1b* much longer than the other coxalae, *2b_1_* subequal *3b_1_*, *2b_2_* and *3b_2_* subequal, *2b_1_* and *3b_1_* longer than *2b_2_* and *3b_2_*, respectively (Table 2). Dorsum of coxa I with a peg-like supracoxal seta (*elc* I) (Fig. 1C).

Gnathosoma (Fig. 2B, C). Dorsal view of the cheliceral base punctated. One pair of galealae (*cs*) and two pairs of hypostomalae (*as* and *bs*) nude; *bs* longer than *cs* and much longer than *as* (Table 2). Hypostomal lip with fimbriation. Palpfemur and palpgenu each with one barbed, pointed dorsal seta (PaScFed and PaScGed). Palptibia with three barbed setae, one on ventral surface, odontus bifid. Palptarsus with seven setae, five nude, one solenidion (ω) and one eupathidium (ζ). fPp = 0-B-B-3B_2_-5Nωζ. Palpal supracoxal seta (*elcp*) peg-like.

Legs (Figs 1B, 3). With seven segments (femora divided). IP = 2533–2692 (Holotype and six paratypes). Claws hook-like and posterior claw with few ciliations, and empodium claw-like. Normal setae on legs barbed and pointed. Leg setal formula: leg I: Cx—1n; Tr—1n; Bfe—4n; Tfe—5n; Ge—1σ, 1κ, 12n; Ti—2φ, 1κ, 1Cp, 18n; Ta—1ω, 1ε, 2ζ, 1Cp, 27n. leg II: Cx—2n; Tr—1n; Bfe—4n; Tfe—5n; Ge—1κ, 12n; Ti—2φ, 19n; Ta—1ω, 1ζ, 28n. leg III: Cx—2n; Tr—1n; Bfe—2n; Tfe—5n; Ge—12n; Ti—1φ, 19n; Ta—1ζ, 28n. The morphometric data of the legs is listed in Table 2.

##### Etymology.

The new species’ name is derived from Taian Town, where the holotype and paratype were collected.

##### Material examined.

***Holotype*.** China • a larva (1087–0275-YN-yl); Yunnan Province, Yulong County, Taian Town; 26°37'8"N, 100°02'9"E; 2502 m; 8 Aug. 2021; Yan Jiang leg.; from *Atkinsoniella* sp. (Hemiptera, Cicadellidae). ***Paratypes*** China • one larva (1088–0275-YN-yl), same data as the holotype. China • three larvae (1082–0271-YN-yl, 1083–0271-YN-yl, 1084–0271-YN-yl); Yunnan Province, Yulong County, Taian Town; 26°37'8"N, 100°02'9"E; 2502 m; 8 Aug. 2021; Yan Jiang leg.; from unidentified *Atkinsoniella* (Hemiptera: Cicadellidae). China • one larva (1085–0272-YN-yl); Yunnan Province, Yulong County, Taian Town; 26°37'8"N, 100°02'9"E; 2502 m; 8 Aug. 2021; Yan Jiang leg.; from an unknown nymph of Cicadellidae. China • one larva (1086–0273-YN-yl); Yunnan Province, Yulong County, Taian Town; 26°37'8"N, 100°02'9"E; 2502 m; 8 Aug. 2021; Yan Jiang leg.; from an unidentified nymph of Cicadellidae.

The holotype and paratypes are deposited in the Institute of Entomology, Guizhou University, Guiyang, China (GUGC).

##### Remarks.

Based on the description of larvae, the genus *Caeculisoma* includes 13 species so far; of them, four species are from Australia and four from China, two species are from Brazil, while the remaining three species are found in Iran, the Republic of South Africa and New Zealand, respectively (Mąkol and Wohltmann 2012; Xu et al. 2020; Kohansal et al. 2024; Noei et al. 2024).

Similar species to *C.taianensis* sp. nov. are currently known as *C.darwiniense* Southcott, 1961, *C.mouldsi* Southcott, 1988, *C.pouyani* Noei & Kohansal, 2024 and *C.sparnoni* Southcott, 1972 based on the key to species of *Caeculisoma* in Kohansal et al. (2024).

The new species differs from *C.darwiniense* by the shape of ASE (nude vs barbed), posterior hypostomalae (*bs*) (nude vs barbed), galealae (*cs*) (nude vs barbed), the number of normal setae on fn Ge I–III (12-12-12 vs 11-12-13), the number of normal setae on fn Ti I–III (18-19-19 vs 17-18-20), longer L (103–114 vs 87–88), Ti I (188–207 vs 102), and Ti III (264–287 vs 143); differs from *C.mouldsi* by the shape of ASE (nude vs barbed), shape of posterior hypostomalae (nude vs barbed), gnathosoma with two pairs hypostomalae (vs one pair hypostomalae), the number of normal setae on fn Ge I–III (12-12-12 vs 12-11-12), the number of normal setae on fn Ti I–III (18-19-19 vs 18-19-18), longer *1b* (64–74 vs 37–58), PaScFed (73–85 vs 42), leg II (800–855 vs 750), leg III (952–1010 vs 915), IP (2533–2692 vs 2455); differs from *C.pouyani* by the shape of ASE (nude vs barbed), galealae (nude vs barbed), hypostomalae (nude vs barbed), palp tarsus with five nude normal setae (vs with five barbed normal setae), longer W (97–108 vs 75–87), *1b* (64–74 vs 40–46), Ti I (188–207 vs 85–95), Ti II (194–216 vs 85–92), Ti III (264–287 vs 115–130), IP (2533–2692 vs 1360–1505) and differs from *C.sparnoni* by shape of ASE (nude vs barbed), cheliceral bases without striations (vs with lengthwise striations), longer L (103–114 vs 83), W (97–108 vs 85), *1b* (64–74 vs 28), Ta I (150–160 vs 77), Ti I (188–207 vs 83), Ta III (165–176 vs 79), Ti III (264–287 vs 100), IP (2533–2692 vs 1220).

The differences between the new species and the four present species of *Caeculisoma* found in China are as follows: *C.taianensis* sp. nov. differs from *C.allopenlineatus* by the positions of ASE (ASE closer to ML than PL vs ASE closer to PL than ML), shape of scutum (pentagonal vs oval), ASE (nude vs barbed), the longer Ti I (188–207 vs 164–170), Ti II (194–216 vs 151–161), Ti III (264–287 vs 227–239), IP (2533–2692 vs 2061–2115), the shorter ML (46–51 vs 114–120); differs from *C.hunanica* by the number of solenidia on Ti II (2 vs 1), the number of normal setae on TFe III (5 vs 4), the number of normal setae on fn Ti I–III (18-19-19 vs 16-16-18), longer leg I (781–834 vs 655), leg II (800–855 vs 621), leg III (952–1010 vs 728), IP (2533–2692 vs 2004); differs from *C.penlineatus* by ASE base location (closer to ML than PL vs in line with the level of PL), BFe I and II with four barbed setae (vs with three barbed setae and one nude seta), palptarsus with one eupathidium (vs with two eupathidia), longer Ti I (188–207 vs 143–167), Ti II (194–216 vs 150–179), Ti III (264–287 vs 213–239), leg I (781–834 vs 627–709), leg II (800–855 vs 645–724), leg III (952–1010 vs 768–878), IP (2533–2692 vs 2060–2298), data based on Xu et al. (2019b, 2020) and differs from *C.semispinus* by the shape of ASE (nude vs with barbs on distal halves), palptibia with three barbed setae (vs with one barbed seta and two nude setae), palptarsus with five nude setae (vs with two barbed setae and three nude setae), longer L (103–114 vs 78–84), W (97–108 vs 71–80), ISD (56–63 vs 41–44), Ti I (188–207 vs 149–163), Ti III (264–287 vs 213–227), IP (2533–2692 vs 2041–2100).

**Figure 4.**
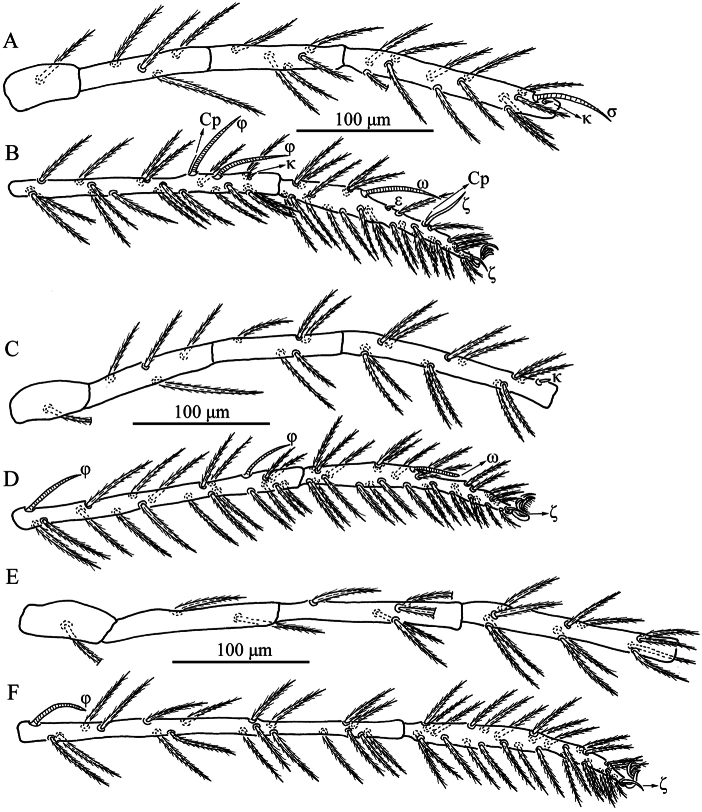
*Caeculisomataianensis* sp. nov., larva. Leg I **A** trochanter—genu **B** tibia—tarsus; leg II **C** trochanter—genu **D** tibia—tarsus; leg III **E** trochanter—genu **F** tibia—tarsus.

### ﻿*Iguatonia* Haitlinger, 2004

#### ﻿ Iguatonia barboproxima sp. nov.

Taxon classificationAnimaliaTrombidiformesErythraeidae

BB63528E-EA2F-5BBD-B8AD-E7FFC9B27022

https://zoobank.org/B7AA6D5B-ADD7-4F98-9245-1789A345EFD3

Figs 5 , 6 , 7 , 8

##### Diagnosis (larva).

ASE and PL located in posterior half of scutum; ASE and PSE with fine barbs on distal halves; two pairs hypostomalae barbed; ISD 30–42.

##### Description.

**Figure 5.**
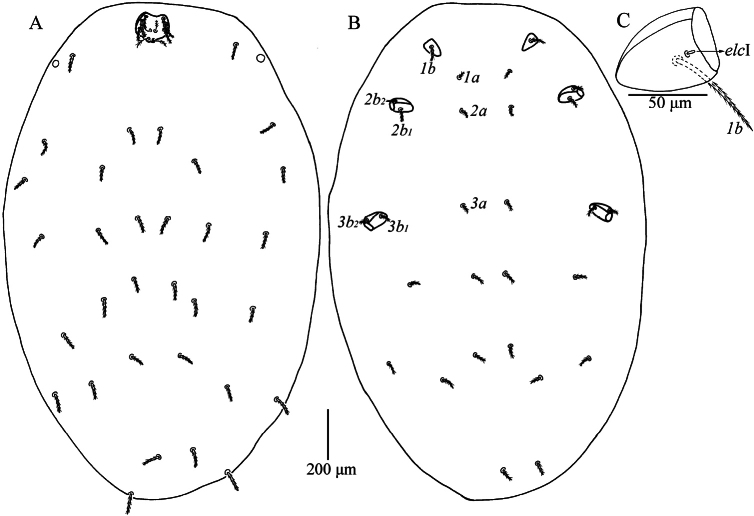
*Iguatoniabarboproxima* sp. nov., larva **A** dorsal view of idiosoma **B** ventral view of idiosoma **C** dorsal view of coxa I, showing supracoxal seta.

**Figure 6.**
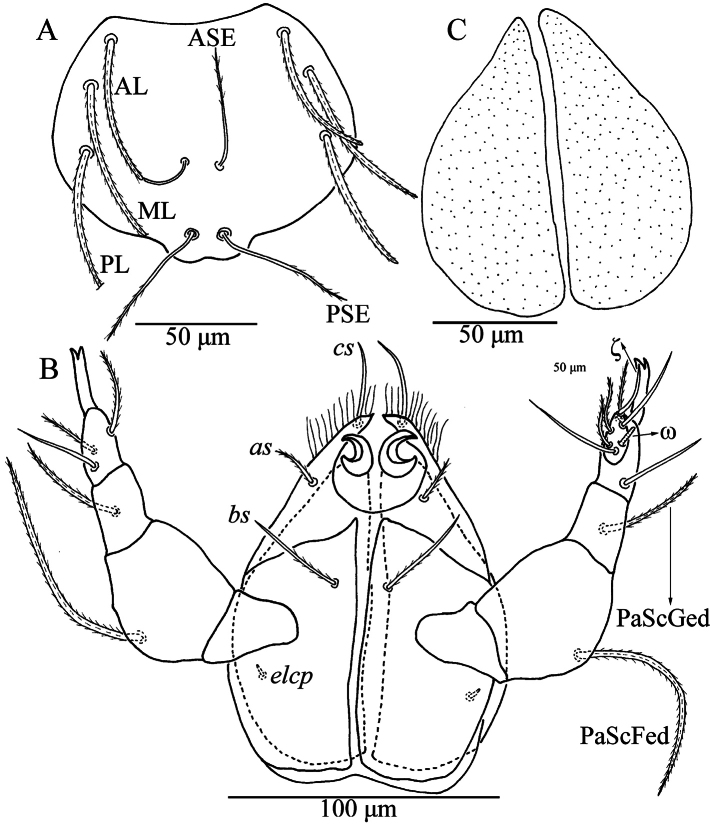
*Iguatoniabarboproxima* sp. nov., larva **A** scutum **B** ventral view of gnathosoma **C** dorsal view of the cheliceral bases.

**Figure 7.**
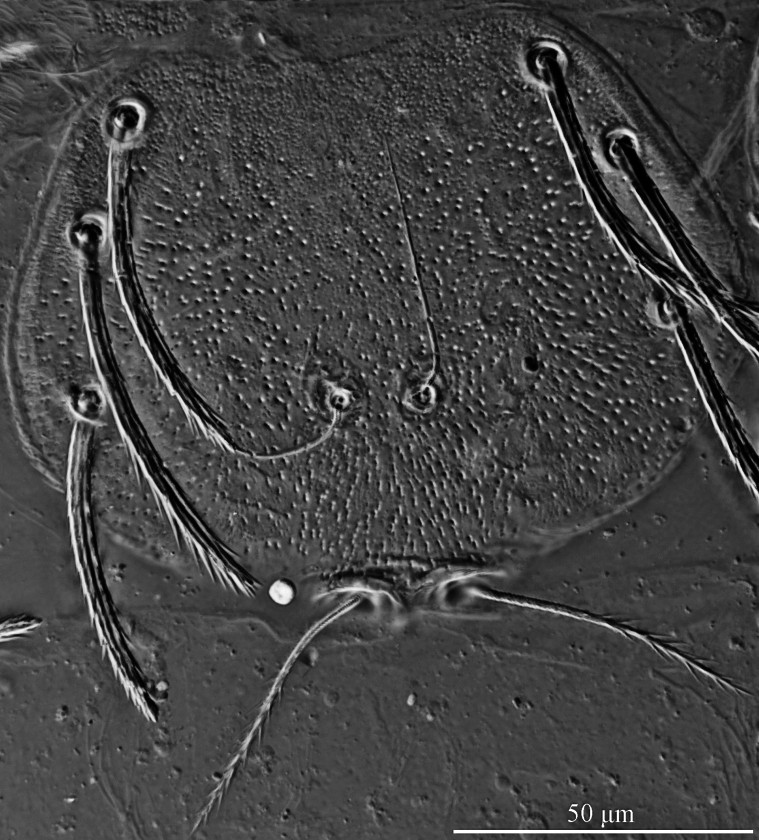
*Iguatoniabarboproxima* sp. nov., larva photograph. Scutum, showing the shape of scutum, ASE and PSE.

Idiosoma almost oval, with 32 (fD = 32–34 in paratypes) barbed setae, a pair of setae located between scutum and eyes at level with PSE bases (Fig. 5A). Scutum about trapezoid outline with rounded angles, wider than long, anterior margin concave, lateral margins arcuate obviously, posterior margin convex in median and with small concave between bases of PSE (Figs 6A, 7). Scutum with three pairs of normal setae (AL, ML and PL) and two pairs of sensilla (ASE and PSE). AL, ML and PL completely barbed, AL slightly shorter than PL, and ML longer than both, PW > MW > AW (Table 3). ASE and PSE with setules in distal half, ASE bases posterior to PL bases, PL placed in posterior half of scutum, PSE near posterior border of scutum and longer than ASE (Fig. 6A, 7).

**Table 3.** Metric and some meristic data of *Iguatoniabarboproxima* sp. nov. (larvae, a–c = paratypes; a: 2024–1767-CQ-wx, b: 2025–1767-CQ-wx, c: 2026–1768-CQ-wx).

Character	Holotype	a	b	c	SD	range
fD	32	34	32	32	0.87	32–34
fV	12	12	12	12	0.00	12–12
NDV	44	46	44	44	0.87	44–46
IL	1928	1998	1672	1733	134.32	1672–1998
IW	1213	1320	1366	1276	56.45	1213–1366
DS	46–77	42–70	47–78	48–77	2.28–3.20	42–78
DS min.	46	42	47	48	2.28	42–48
DS max.	77	70	78	77	3.20	70–78
PDS	54–77	53–70	51–78	52–77	1.12–3.20	51–78
PDS min.	54	53	51	52	1.12	51–54
PDS max.	77	70	78	77	3.20	70–78
Oc	20	20	21	23	1.22	20–23
*1a*	24	28	25	26	1.48	24–28
*2a*	32	37	34	37	2.12	32–37
*3a*	33	31	34	34	1.22	31–34
*1b*	54	63	55	65	4.82	54–65
*2b_1_*	42	40	41	44	1.48	40–44
*2b_2_*	40	39	40	39	0.50	39–40
*3b_1_*	40	39	40	42	1.09	39–42
*3b_2_*	34	34	37	36	1.30	34–37
L	103	100	109	106	3.35	100–109
W	124	123	137	130	5.59	123–137
AW	72	70	81	77	4.30	70–81
MW	91	88	98	97	4.15	88–98
PW	99	98	109	104	4.39	98–109
MA	60	59	63	62	1.58	59–63
AA	14	15	16	16	0.83	14–16
SB	14	14	18	17	1.79	14–18
ISD	30	34	42	37	4.38	30–42
AP	47	47	56	60	5.68	47–60
AL	56	53	61	59	3.03	53–61
ML	70	68	76	71	2.95	68–76
PL	59	57	64	62	2.69	57–64
ASE	44	46	46	45	0.83	44–46
PSE	55	59	66	61	3.96	55–66
*as*	18	16	15	20	1.92	15–20
*bs*	37	35	37	41	2.18	35–41
*cs*	32	34	31	35	1.58	31–35
PaScFed	81	76	77	80	2.06	76–81
PaScGed	41	37	46	40	3.24	37–46
GL	136	146	137	141	3.94	136–146
Ta I (H)	18	19	19	19	0.43	18–19
Ta I (L)	159	154	147	157	4.55	147–159
Ti I	167	166	163	166	1.50	163–167
Ge I	135	133	131	135	1.66	131–135
TFe I	83	82	78	78	2.28	78–83
BFe I	89	90	89	91	0.83	89–91
Tr I	50	51	56	51	2.35	50–56
Cx I	73	66	67	63	3.63	63–73
Ta II (H)	19	16	18	18	1.09	16–19
Ta II (L)	150	147	143	152	3.39	143–152
Ti II	178	169	165	174	4.92	165–178
Ge II	132	130	127	132	2.05	127–132
TFe II	74	74	72	79	2.59	72–79
BFe II	91	86	88	89	1.80	86–91
Tr II	60	56	57	57	1.50	56–60
Cx II	81	86	78	79	3.08	78–86
Ta III (H)	17	16	17	19	1.09	16–19
Ta III (L)	165	164	154	163	4.39	154–165
Ti III	252	251	246	254	2.95	246–254
Ge III	150	144	139	147	4.06	139–150
TFe III	115	117	118	111	2.68	111–118
BFe III	117	120	121	114	2.74	114–121
Tr III	67	61	56	54	5.02	54–67
Cx III	80	86	86	77	3.90	77–86
Leg I	756	742	731	741	8.90	731–756
Leg II	766	748	730	762	14.10	730–766
Leg III	946	943	920	920	12.30	920–946
IP	2468	2433	2381	2423	31.01	2381–2468

Venter. All ventral setae, including coxalae, barbed and with pointed ends (Fig. 5B). Dorsum of coxa I with a peg-like supracoxal seta (*elc* I) (Fig. 5C). Three pairs of intercoxal setae (*1a*, *2a* and *3a*), *1a* posterior to level of posterior edge of coxae I, *2a* between coxae II, and *3a* at a line with anterior edges of coxae III. *2a* and *3a* subequal and both slightly longer than *1a* (Table 3). Five pairs of coxalae (*1b*, *2b_1_*, *2b_2_*, *3b_1_* and *3b_2_*), *1b* longest, *2b_1_*, *3b_1_*, and *2b_2_* subequal and all slightly longer than *3b_2_* (Table 3). 12 setae behind coxae III (fV = 12 in paratypes).

Gnathosoma (Fig. 6B) with a pair of nude galealae (*cs*), two barbed anterior hypostomalae (*as*) and two posterior hypostomalae with barbs on proximal half, *bs* slightly longer than *cs*, and both longer than *as* (Table 3). Hypostomal lip fimbriated. Cheliceral bases punctate on the dorsal surface (Fig. 6C). Palpfemur and palpgenu, each with one barbed, pointed dorsal seta. Palptibia with one nude ventral seta, one barbed ventral seta, and one barbed dorsal seta, odontus bifid. Palptarsus with seven setae, three barbed, two nude, one solenidion and one eupathidium. fPp = 0-B-B-2BN_2_-3B2Nωζ. Palpal supracoxal seta (*elcp*) peg-like.

**Figure 8.**
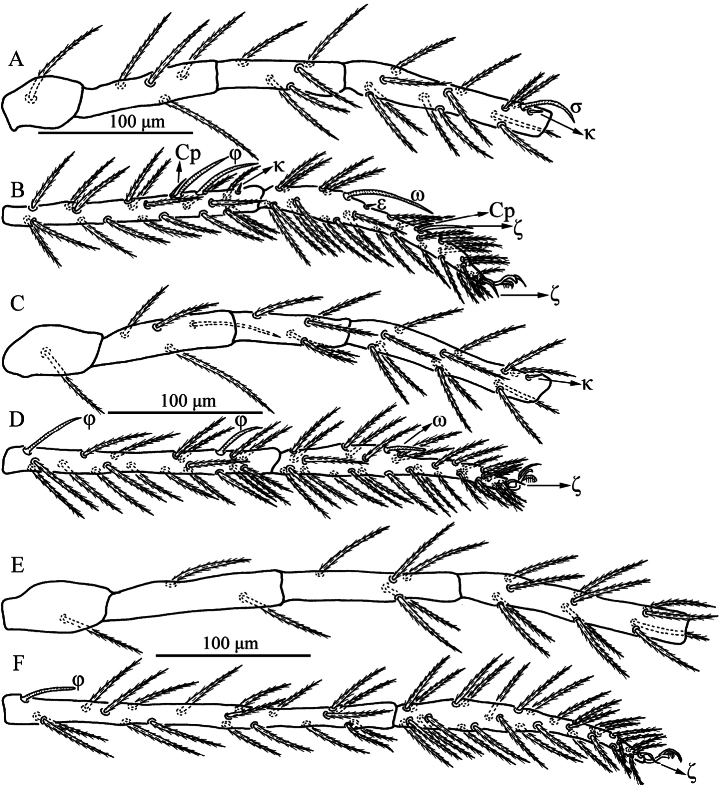
*Iguatoniabarboproxima* sp. nov., larva. Leg I **A** trochanter—genu **B** tibia—tarsus; leg II **C** trochanter—gen **D** tibia—tarsus; leg III **E** trochanter—genu **F** tibia—tarsus.

Legs (Figs 5B, 8) with seven segments (femora divided). IP = 2381–2468 (Holotype and three paratypes) (Table 3). Anterior and posterior claws hook-like, subequal in length, and anterior claw with few ciliations. Claw-like empodium falciform, longer and slenderer than lateral claws. Normal setae on legs barbed and pointed. Leg setal formula: Leg I: Cx—1n; Tr—1n; Bfe—4n; Tfe—5n; Ge—1σ, 1κ, 12n; Ti—2φ, 1κ, 1Cp, 18n; Ta—1ω, 1ε, 2ζ, 1Cp, 29n. leg II: Cx—2n; Tr—1n; Bfe—4n; Tfe—5n; Ge—1κ, 12n; Ti—2φ, 19n; Ta—1ω, 1ζ, 30n. leg III: Cx—2n; Tr—1n; Bfe—2n; Tfe—5n; Ge—12n; Ti—1φ, 19n; Ta—1ζ, 30n.

##### Etymology.

The specific epithet of the new species refers to the posterior hypostomalae, which exhibit fine barbs on their proximal half.

##### Material examined.

***Holotype*** China • a larva (2023–1767-CQ-wx); Chongqing Municipality, Wuxi County, Shuangyang Town; 31°31'29"N, 109°50'12"E; 1151 m; 30 Jun. 2022; Yan Jiang leg.; from an unidentified nymph of Cicadellidae (Hemiptera). ***Paratypes*** China • two larvae (2024–1767-CQ-wx, 2025–1767-CQ-wx), the same data as the holotype. China • one larva (2026–1768-CQ-wx); Chongqing Municipality, Wuxi County, Shuangyang Town; 31°29'28"N, 109°49'44"E; 1132 m; 30 Jun. 2022; Xiao-Li Xu leg.; from an unidentified nymph of Cicadellidae (Hemiptera).

The holotype and paratypes are deposited in the Institute of Entomology, Guizhou University, Guiyang, China (GUGC).

##### Remarks.

To date, two species of *Iguatonia* have been documented based on larvae, one from Brazil and another one from China (Haitlinger 2004; Noei et al. 2024; Xu et al. 2020).

*Iguatoniabarboproxima* sp. nov. differs from *I.barbillae* by the shape of scutum (about trapezoid vs quadrilateral), PL location (in posterior half of scutum vs in anterior half of scutum), ASE location (closer to PL than PSE vs far from PL and near PSE), longer ISD (30–42 vs 10), Ti I (163–167 vs 76–78), and Ti III (246–254 vs 116–126) and differs from *I.xinfengi* by the shape of scutum (about trapezoid vs sub-rounded), shape of hypostomalae (barbed vs nude), positions of ASE (far from PL and closer to PL than PSE vs almost at the same line with PL), longer ISD (30–42 vs 20–21), Ti I (163–167 vs 114–117), and Ti III (246–254 vs 194–197).

## ﻿Discussion

Considering previously published data and the present study, 13 species of *Caeculisoma* have been documented based on the larval stage and one species was recorded based on both the larval and post-larval instars. Of them, only five species (*C.brazilensis* Noei & Šundić, 2024; *C.carmenae* Haitlinger, 2008; *C.nestori* Haitlinger, 2004; *C.hunanica* Zheng, 2002; and *C.sparnoni* Southcott, 1972) are without host records (Southcott 1972; Zheng 2002b; Haitlinger 2004, 2008; Kohansal et al. 2024; Noei et al. 2024). The hosts of *Caeculisoma* larvae were recorded in Insecta, comprising three orders (Lepidoptera, Orthoptera, Hemiptera), and seven families (Acrididae, Cicadellidae, Cicadidae, Delphacidae, Geometridae, Issidae, Miridae) (Southcott 1961, 1972, 1988; Stroiński et al. 2013, Xu et al. 2019a, b, 2020; Kohansal et al. 2024). Only *C.pouyani* Noei & Kohansal, 2024 has a host distribution across three families (Acrididae, Cicadellidae, Miridae) in two orders (Hemiptera, Orthoptera); and *C.penlineatus* Xu & Jin, 2019 has a host distribution across three families (Cicadellidae, Delphacidae and Issidae). And the host of each of the remaining species was only recorded in a single family: three species (*C.allopenlineatus* Xu & Jin, 2020; *C.semispinus* Xu & Jin, 2019; *C.taianensis* sp. nov.) with hosts were recorded in Cicadellidae (Hemiptera), two species (*C.cooremani* Southcott, 1972; *C.darwiniense* Southcott, 1961) with hosts were recorded in Acrididae (Orthoptera), *C.mouldsi* Southcott, 1988 with a host was recorded in Cicadidae (Hemiptera), and *C.huxleyi* Southcott, 1972 with a host was recorded in Geometridae (Lepidoptera). According to the available data, there is a higher probability of finding larvae of the genus *Caeculisoma* ectoparasitic on Hemiptera compared to Orthoptera and Lepidoptera.

*Iguatoniabarbillae* Haitlinger, 2004 from an unidentified Homoptera insect (Hemiptera), *I.xinfengi* Xu & Jin, 2020 from an unknown Delphacidae (Hemipteran) and *I.barboproxima* sp. nov. from an unidentified Cicadellidae species (Hemiptera) indicate that the host’s spectrum of *Iguatonia* species is limited to the order Hemiptera.

Only three families of Hemiptera, including Cicadellidae, Delphacidae and Issidae were documented in China for *Caeculisoma* hosts. A similar situation occurs in the genus *Iguatonia*, whose hosts are from two families (Cicadellidae, Delphacidae) of Hemiptera.

According to the present information of these two genera the hosts are limited, which may be related to the locations, times, and methods of collection. Therefore, in future studies of their taxonomy, more attention should be paid to geographical ranges and the use of diverse collection methods at different periods in order to know more about the host.

## Supplementary Material

XML Treatment for Abrolophus quadrapexicis

XML Treatment for Caeculisoma taianensis

XML Treatment for Iguatonia barboproxima

## ﻿Additional information

### Author ORCIDs

Yan Jiang https://orcid.org/0000-0002-8980-5344

Tian-Ci Yi https://orcid.org/0000-0002-9953-3709

Si-Yuan Xu https://orcid.org/0000-0001-6467-6120

Dao-Chao Jin https://orcid.org/0000-0003-2727-5621

### Data availability

All of the data that support the findings of this study are available in the main text.
